# Current and Future Applications of AI in EMS Training: A Scoping Review

**DOI:** 10.7759/cureus.90469

**Published:** 2025-08-19

**Authors:** Logan D Pasquariello, Jessica K Sims, Jordan O'Brien, Jeffrey Upperman

**Affiliations:** 1 Pediatric Surgery, Vanderbilt University Medical Center, Nashville, USA; 2 Epidemiology, Columbia University Mailman School of Public Health, New York, USA

**Keywords:** artificial intelligence, disaster medicine, emergency medical services, ems education, large language models, machine learning, personalized learning, prehospital care, simulation training, virtual reality

## Abstract

Over the last 15 years, AI has been increasingly utilized in healthcare education. In EMS, AI is being used to train providers for their high-stress, dynamic environments. This scoping review examines current and future applications of AI in EMS training, with a focus on simulation, interventions, personalized learning, and disaster preparedness. A librarian-assisted literature search was conducted across PubMed, Embase, and Web of Science using terms related to EMS, AI, and education. Articles published between January 1, 2010 and March 16, 2025 were screened and selected based on relevance. Studies were reviewed by title, abstract, and full text, followed by data extraction. Currently, AI has been integrated to enhance EMS training through advanced simulation, procedure training, development of personalized learning materials, and disaster response. These improvements are made possible by utilizing different types of AI tools such as machine learning, natural language processing, and large language models. AI tools showed improvements in realism, diagnostic accuracy, feedback delivery, and learner adaptability. It is actively transforming EMS training by strengthening provider readiness, clinical judgment, and educational outcomes. With continued research and development, its integration could further enhance training effectiveness. Ethical and implementation challenges remain, but increased education and awareness can help ensure that AI is used to positively affect patient care.

## Introduction and background

AI refers to the ability of technology to learn, interpret data, and complete tasks that typically require human input [1]. AI is an umbrella term for large language models (LLMs), natural language processing (NLP), and machine learning (ML) [2-4]. The healthcare sector is rapidly adopting AI because of its ability to generate and/or synthesize large quantities of data quickly [2,4,5]. This adaptability is especially useful in dynamic environments like EMS [6]. In EMS, emergency medical technicians, paramedics, and sometimes other healthcare professionals deliver prehospital care to sick and injured patients.

EMS personnel go through extensive training due to the vast possibilities of emergencies they could be called upon to handle. Training these professionals inherently presents challenges because it is difficult to plan for every emergency. Research has shown that simulation-based education provides a more accurate representation of real high-stress environments [7,8]. In current practice, EMS departments use one or a combination of high-fidelity mannequins, virtual/augmented/mixed reality, and online simulations to train both new and seasoned providers. Aside from education, AI has been thoroughly researched due to its promising application in aiding EMS triage decisions, dispatch, data collection of electronic patient care reports, and diagnostic capabilities [6,9,10].

Current AI applications are increasingly addressing specific EMS training needs, such as improving procedural skills, enhancing communication, and preparing for disaster scenarios. Practical examples include AI-enhanced simulations and adaptive learning tools that offer tailored training experiences.

This review aims to examine how AI is currently applied in EMS education, identify future opportunities, and discuss research gaps.

## Review

Methods

A literature search was conducted using terms related to AI applications in EMS training. PubMed, Web of Science, and Embase were searched, and results were imported into Covidence for screening. A librarian assisted in designing the search terms to ensure comprehensive coverage (Appendix A, Appendix B, Appendix C). Searches were restricted to the period from 2010 to the present to capture the contemporary use of AI.

**Figure 1:**
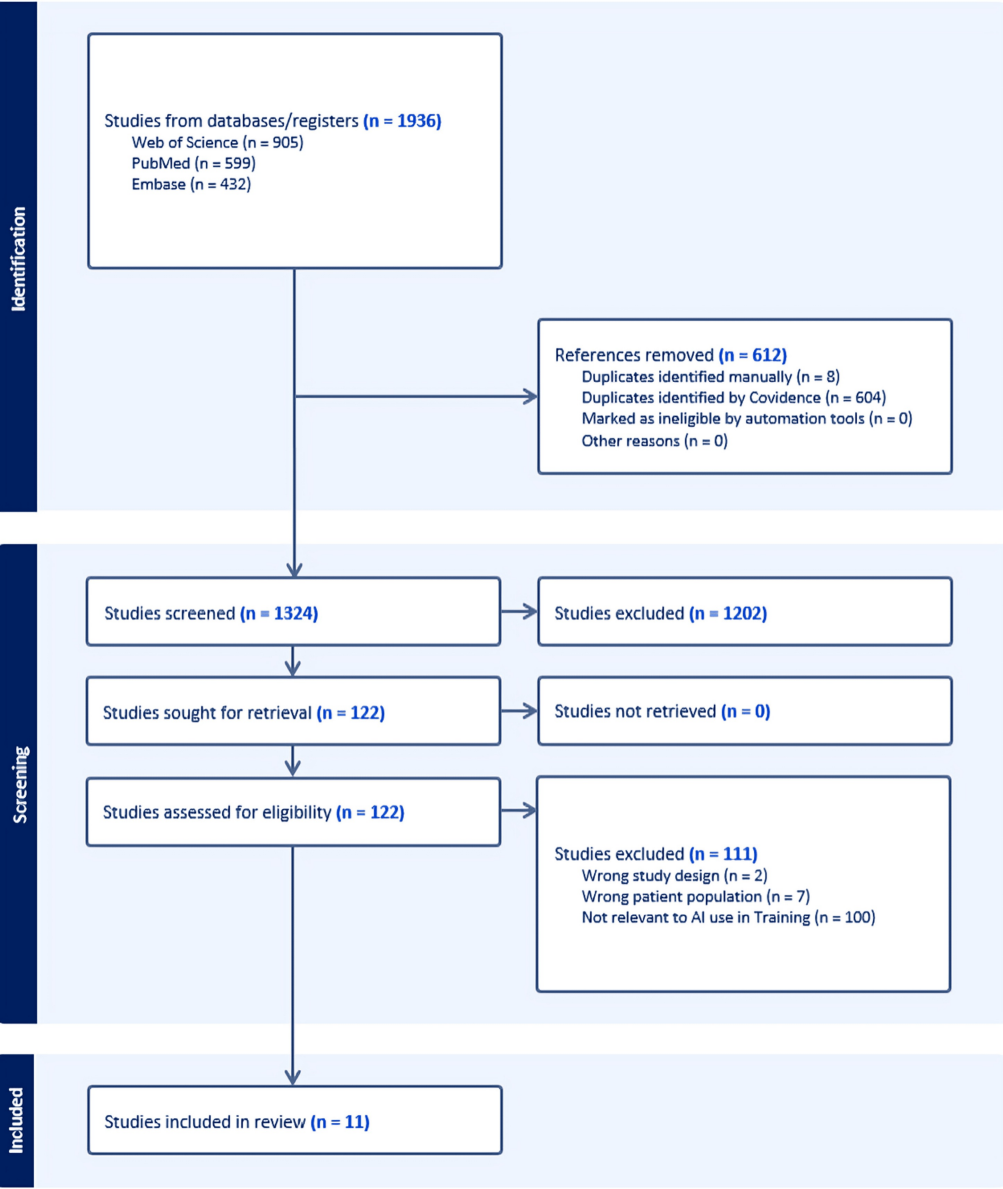
Flow diagram showing the screening process

Inclusion criteria were EMS-specific scientific articles addressing the study objectives, written in English, and published between January 1, 2010, and March 16, 2025. Exclusion criteria were literature that did not address the role of AI in EMS education or was published before 2010. Studies were considered EMS-specific if they directly involved EMS personnel, replicated prehospital environments, or validated interventions in EMS settings. Articles were screened by title and abstract, followed by full-text review, and relevant data were extracted. No formal statistical analysis was performed due to variability among studies; instead, the review provides a descriptive and thematic synthesis of findings. The screening process is outlined in Figure 1.

The core search terms included combinations of keywords related to EMS personnel (emergency medical technicians, paramedics, first responders), AI technologies (artificial intelligence, machine learning, large language models, automated reasoning), immersive technology (virtual, augmented, and mixed reality), and educational contexts (training, simulation, education, literacy, workshop).

Results

The studies identified in this review present a variety of applications for AI in EMS training. These include enhanced simulations, improved clinical decision-making, and personalized education strategies. Table 1 outlines key takeaways from selected studies.

**Table 1:** Key findings of the studies included in the review AR, augmented reality; KNN, k-nearest neighbors; LLM, large language model; ML, machine learning; NLP, natural language processing; NN, neural network; PTSD, post-traumatic stress disorder; SVM, support vector machine

Authors	Study title	Key takeaway	Reference
Oregui et al. (2024)	Augmented reality interface for adverse-visibility conditions validated by first responders in rescue training scenarios	ML-based technology, such as the Robust Vision Model, that integrates into AR, helps emergency responders detect significant objects in low-visibility rescue scenarios, improving training and real-world disaster response.	[11]
Javanbakht et al. (2024)	Unreal that feels real: artificial intelligence-enhanced augmented reality for treating social and occupational dysfunction in post-traumatic stress disorder and anxiety disorders	AI-integrated AR provides exposure therapy for EMS personnel dealing with PTSD, creating realistic, interactive scenarios to enhance mental readiness.	[12]
Gutiérrez Maquilón et al. (2024)	Integrating GPT-based AI into virtual patients to facilitate communication training among medical first responders: usability study of mixed reality simulation	AI-driven generative voice agents like ChatGPT improve communication training for first responders. Computational demands and response delays present challenges.	[13]
Grosjean et al. (2024)	Digital health education for the future: the SaNuRN (Santé Numérique Rouen-Nice) consortium’s journey	The SaNuRN program integrates AI and NLP into virtual clinical simulators, allowing EMS trainees to experience both provider and patient perspectives for better communication and care.	[14]
Carlson et al. (2016)	A novel artificial intelligence system for endotracheal intubation	AI models (KNN, SVM, Decision Trees, and NN) analyze video recordings to improve EMS endotracheal intubation training, with real-time feedback enhancing skill development.	[15]
Cheng et al. (2021)	Deep learning assisted detection of abdominal free fluid in Morison’s pouch during focused assessment with sonography in trauma	A deep learning model (ResNet50-V2) accurately classifies ultrasound images for EMS personnel, improving diagnostic accuracy and efficiency in trauma assessments.	[16]
Park and Sung (2024)	Automated surgical wound classification for intelligent emergency care applications	AI models (ResNeXt-101 32x8d and ViT) classify wound images with high accuracy, providing real-time treatment recommendations and improving EMS coordination with the receiving hospital	[17]
Vrdoljak et al. (2025)	A review of large language models in medical education, clinical decision support, and healthcare administration	LLMs like ChatGPT can generate and evaluate medical questions to help fill knowledge gaps in medical education. They are also useful in providing the most up-to-date medical protocols and practices.	[18]
Mehta et al. (2022)	Human-centered intelligent training for emergency responders	The LEARNER system personalizes EMS training using AI-powered physiological and behavioral markers, enhancing learning outcomes through adaptive education.	[19]
Ogundiya et al. (2024)	Looking back on digital medical education over the last 25 years and looking to the future: narrative review	AI, particularly LLMs like ChatGPT, aids in medical education by generating case scenarios, explanations, and study materials tailored to individual learners.	[20]
Kao et al. (2023)	The development of new remote technologies in disaster medicine education: a scoping review	Machine learning (Federated Learning) enhances disaster medicine education, offering decentralized, real-time training models for EMS personnel to improve disaster response.	[21]

Enhancing Virtual Reality (VR) Simulations and Patient Care

VR, augmented reality (AR), mixed reality (MR), and other smart technologies are increasingly being used in EMS education to simulate real-world emergencies. Recent advancements have led to AI’s integration into these technologies. The research has shown that AI can often enhance these immersive experiences in situations including rescue training, psychological preparedness, and communication skills through virtual patients’ simulations. The following papers talk about specific details regarding its integration and show that they enhance realism, adaptability, and effectiveness in EMS education.

A study by Oregui et al. examined the implementation of AR technology for limited visibility conditions during rescue scenarios [11]. The ML model, called the Robust Vision Model, helps aid responders in detecting relevant objects, such as casualties, by interpreting information from a thermal camera feed. These technologies were validated during rescue training scenarios and present a significant potential to be further refined into a marketable product. These findings suggest that the technology holds promise for future integration into EMS training and operational deployment in disaster and rescue contexts.

Next, a study conducted by Javanbakht et al. explored the use of AI-integrated AR to help first responder (FR) clinicians deal with PTSD and other fear- and trauma-related conditions [12]. FRs, such as EMS personnel, experience a lot that can lead to developing these conditions, so it can be beneficial to allow them to practice patient encounters. In this model, ML is used in AR simulations to develop realistic characters and have unscripted conversations with a realistic tone of voice. ML also allows virtual characters to respond quickly in about one second, further enhancing realism. The therapist can also change all these features based on the patient’s needs. Patients found that this exposure therapy can often act just as triggering as real environments, which can positively contribute to their treatment. Ultimately, this AI-enhanced technology allows FRs to simulate real environments that can further train them in operating with poise under pressure.

Similarly, Gutiérrez Maquilón et al. tested AI-integrated virtual patients to facilitate better communication between providers and patients [13]. This study aimed to determine the usability of generative voice agents (GVAs), such as ChatGPT, to communicate with medical FRs (MFRs) and to evaluate the overall realistic perception of the technology. It was shown that ChatGPT successfully integrated into their mixed-reality training scenario to facilitate communication and provide unique feedback that MFRs would not get with a scripted interaction. However, the study identified some challenges with implementation. ChatGPT models are computationally expensive, requiring numerous parameters and adjustments. They also occasionally manufacture false information and have a noticeable delay (three seconds) between questions and responses. These, however, were identified as fixable with further research. Overall, MFRs surveyed showed that they had a moderately high perception of the naturalness of the GVA’s voice quality and an equal likeability perception for the GVA’s usability.

The SaNuRN program, reviewed by Grosjean et al., further demonstrates the educational potential of AI in simulated training [14]. This program has a section that utilizes AI, specifically NLP, in a virtual clinical simulator. The AI enables the program to act as either the patient or the professional, providing questions and responses accordingly. This trains providers in a low-stakes environment and allows them to view both perspectives, which can be important in a job like EMS, where you are often not aware of how your assessment may appear to the patient.

Together, these studies illustrate how AI-enhanced virtual environments can extend the scope of EMS training by making simulations more immersive, emotionally engaging, and responsive to learner behavior.

AI-Assisted Intervention and Assessment Training

Some EMS providers are trained in complex interventions such as endotracheal intubation (ETI), point-of-care ultrasound (POCUS), and wound classification. These skills are hard to learn and even more difficult to routinely practice. Instructors often try to simulate real scenarios, but this is often too expensive and time-consuming. As a result, researchers have developed AI-optimized tools that help identify structures that can lead to successful interventions.

Carlson et al. developed an AI system that assists in ETI training by analyzing video recordings of intubation attempts [15]. The AI in this study uses ML models to analyze video recordings of ETI attempts to detect the glottic opening. The four ML models used were k-nearest neighbor (KNN), support vector machine (SVM), decision trees, and neural networks. They were trained on video data from a mannequin, with half being used as a training set and the other half being used for testing. These models analyzed one-second video intervals to determine the presence or absence of the glottic opening. The results showed that KNN and SVM achieved 80% accuracy, while decision trees and neural networks achieved a slightly lower accuracy. The study identified further implications for the model in providing real-time feedback to providers during intubation attempts. This could enhance training for novice and inexperienced providers who have little experience visualizing certain anatomical structures.

Similarly, Cheng et al. examined how AI can aid in enhancing POCUS training and assessment [16]. This paper applies AI to assist providers with identifying the amount of free fluid within Morrison’s pouch during a focused assessment with sonography in a trauma exam. The AI used is a deep learning (DL) model called Residual Networks 50-Version 2 (ResNet50-V2). The DL model classifies ultrasound images based on the presence or absence and quantity of free fluid. The model was trained using a large dataset of ultrasound images obtained from patients admitted to an emergency department. The images were evaluated by expert clinicians and grouped into categories that were tested against the AI model. The model had highly accurate results, reaching 96% accuracy. The automated feedback system was also able to evaluate image quality and give feedback on whether the ultrasound images were appropriate for detecting free fluid. This AI model could benefit EMS training by enhancing diagnostic accuracy in the pre-hospital setting and allowing for less training on POCUS, potentially making it more accessible in the field. The real-time feedback in the setting of traumatic injury can allow EMS personnel to more accurately inform hospital providers about concern for intra-abdominal hemorrhage, allowing for quicker mobilization of resources, especially if an operation is necessary.

Lastly, Park and Sung examined the use of an AI model to aid EMS in wound classification and treatment recommendations [17]. This AI system consists of a DL model (ResNeXt-101 32x8d) and a Vision Transformer (ViT) to classify various wound types from images and provide feedback. These models were trained on a dataset of wound images to analyze and classify wounds based on several categories, such as shape, cause, and severity. The ViT presented the highest accuracy of 92.78%. This model can benefit EMS training and assessment by providing real-time guidance to providers on how to treat wounds they see less frequently. The system can also improve coordination and communication between in- and prehospital providers by transmitting these images to the hospital before arrival.

These AI-integrated tools provide an important layer of support in training EMS personnel for skilled procedures. They offer timely feedback, enhance diagnostic accuracy, and improve coordination, leading to an overall increase in prehospital care scope and quality.

Generation of Personalized Education Materials

With EMS professionals often coming from various backgrounds, there may be learning gaps within the class. This is why it is important to tailor education to each individual and their respective learning gaps or styles. However, this presents a challenge for many instructors because of large class sizes and limited time or resources available. Due to these challenges, AI, specifically LLMs, is being utilized to help generate personalized educational materials and plans.

Vrdoljak et al. conducted a review on LLMs’ use in the development of case vignettes [18]. The review found that the LLM, ChatGPT, was effective in developing medical questions and evaluating answers. They highlight AI’s ability to provide the most up-to-date medical information but also address challenges such as ChatGPT’s hallucinations, which confidently state inaccurate facts.

Mehta et al. explored the LEARNER system in emergency responder education [19]. This system builds on and uses the AI application, human-augmented technologies. The goal of this system is to personalize training and tailor education materials to individual emergency responders. The LEARNER system utilizes physiological and behavioral (PNB) markers, collected via wearable sensors, self-reports, and interaction behaviors, to tailor the learning curriculum to each individual’s unique educational needs. The framework consists of three core components: actionable measures, adaptable elements, and a guiding strategy. Actionable measures are formed from the real-time PNB markers. Adaptable elements refer to aspects of the training experience that can be modified to address deficiencies or reinforce positive behaviors. The guiding strategy determines how the content is adjusted, based on the learner's responses. Overall, the implementation of the LEARNER system enhances educational effectiveness through an adaptable learning curriculum and addresses some key shortcomings in EMS training.

A 25-year review of literature on digital medical education by Ogundiya et al. examined the current use of AI in developing personalized educational materials [20]. While the studies reviewed encompassed a variety of healthcare professionals, paramedics were among them, making the findings relevant to EMS training contexts. This paper discusses the growing use of LLMs such as ChatGPT to give quick explanations and summaries of long and complex medical scenarios. This can therefore be used to generate unique patient cases and practice questions. These AI systems can also be applied to develop educational curricula and other educational materials specific to the learner. AI text-to-image has also been proven to create useful illustrations and can help visualize certain topics for students struggling with conceptualizing the material.

EMS providers often enter training with different levels of experience, making personalized education important but difficult to achieve. AI tools like LLMs and adaptive learning systems offer a scalable way to tailor materials, improve engagement, and strengthen preparedness.

Improving Education in Disaster Medicine (DM) Management and Preparedness

In a scoping review by Kao et al., they found that ML plays an important role in DM education [21]. They found AI particularly helpful in educating providers on the management and preparedness of disaster scenarios. The authors reviewed the federated learning (FL) approach, which uses ML to efficiently train and update models in disaster scenarios, making it applicable to EMS, a crucial component in pre-hospital DM. The FL approach uses a decentralized data collection technique that trains models without requiring collaboration with a central server. This helps keep the application local, making it more efficient, enabling integration with multiple mobile edge computing systems, and enhancing data privacy. This FL approach also allows training on real disaster datasets to develop models that provide FRs and EMS personnel with realistic training simulations. These models can ultimately be used to educate EMS and disaster response teams on important patterns, how disasters may present, and how to manage the scene effectively.

Discussion

Current Use of AI in EMS Training

AI’s developing role in EMS training is being used to enhance provider preparedness, assessments, and education. In this scoping review, the included studies consistently demonstrated positive impacts on learner engagement, skill acquisition, and confidence. Across the literature, these tools have been shown to increase training opportunities and offer individualized feedback, thereby improving access to training and enhancing simulation realism. AI should be embraced for its potential to enhance provider competency and confidence. However, successful implementation will need to align with existing training applications and be aware of resource disparities across EMS agencies. Few included studies directly compare AI-based training with traditional EMS education, making this an important gap for future research. As adoption grows, future research should also focus on identifying the applications that prove to be the most efficient and cost-effective.

Future Use of AI in EMS Training

As AI technologies continue to evolve, so does their potential to transform EMS training. Several studies identified during screening recognized opportunities for such improvements but did not actually implement AI in their training programs. Baetzner et al. identified that AI would be useful in integrating into their current system to improve efficiency [22]. The system examined in the article was used to enhance mass casualty incident (MCI) training through immersive VR. They suggest that AI could be used in future applications to give trainees real-time feedback to improve their information transmission. They also identify that AI could be used to interpret live data and, in real-time, adjust the scenario difficulty to meet the individual needs of the trainee. Similarly, Zechner et al. examined MCI training through VR and identified challenges that AI may effectively address [23]. In this application, the researchers found that VR limits how many simulated patients can talk at once because of diminishing quality of speech and a lack of resources. AI was identified to be able to enhance realism by improving the interactivity of virtual patients. This would be achieved because AI could be programmed to provide adaptive, context-specific replies that respond to the actions and questions of the providers. Similarly, an article by Ankam et al. discussed the potential use of AI integration into MR in pediatric emergencies to reduce medication errors [24]. They specifically point out AI-based patient condition detection and automatic weight measurement as potential future AI-assisted assessments. An article by Wolcott and English analyzed the current and future state of AI in prehospital stroke care and evaluation [25]. They identified that AI could be used in training providers by providing real-time feedback and offering immediate insights into stroke assessment. They also mention that AI could be useful in integrating VR/AR simulations to provide tailored training to providers. Tharun et al. also examined the use of AR in EMS training; however, they identified AI as a useful tool in estimating blood loss and providing feedback on the best intervention [26]. Birt et al. also identified AI as useful in integrating into mixed reality simulations to track and assist the learner’s performance and skill development [27]. Lastly, a perspective article by Ventura and Denton signified the use of AI in the generation of medical materials to enhance medical education and fill knowledge gaps [9].

These articles demonstrate that AI integration is ever evolving, and academic research may not keep up with the current integration within EMS department training. However, the literature identifies that there is great promise in further application within EMS education.

Ethical and Logistical Considerations

Integrating AI into EMS training presents some challenges. When dealing with AI, it is important to understand and be aware of ethical considerations. EMS, including training, must adhere to the Health Insurance Portability and Accountability Act and similar laws around the world. While training should not include protected information, it may be overlooked when AI is being used. AI can also have unintentional bias when only trained on certain groups of people or types of interventions. There is also a liability factor to be considered. In a very vulnerable-to-liability profession such as EMS, there must be clear guidance on accountability if an AI application were to give faulty recommendations. While most of these challenges can be fixed through thorough education, it is still important to explicitly address them.

With EMS departments notoriously underfunded and understaffed, it is also important to address logistical concerns. Most departments are not technologically advanced and may have trouble running these AI models. There is also a concern about whether it is possible to integrate AI systems into existing training systems. If not, there could be significant costs and resource allocation, and it may not be feasible for some departments. These ethical and logistical barriers parallel challenges encountered in implementing AI-assisted MCI training, where cost, technological capacity, and liability concerns can hinder adoption.

Limitations

While this review aimed to be as thorough as possible, there were some limitations. The authors chose to include studies from 2010 onward, as this was considered a reasonable timeframe given the recent growth of AI. Additionally, only three databases were searched, as they were expected to yield the most relevant results. Grey literature was not included. Because of these choices, some relevant studies may have been missed.

There was also a significant amount of literature covering virtual simulation (VR/AR/MR) education in EMS. Some of these may have used AI to enhance the scenarios. If the authors did not clearly mention using AI or related terms (like ML, NLP, LLM, ChatGPT, etc.), the study was excluded, as it was considered outside the scope of this review.

## Conclusions

AI’s emerging role in EMS training is being used to enhance provider preparedness, assessments, and education. AI-driven models in VR simulations have shown that they enhance realism and gain better results according to trainees. AI-assisted intervention and assessment tools, such as ML for ETI or DL for POCUS, have shown real-time feedback to providers. Additionally, AI-driven personalized learning materials address education gaps between individuals in a class, while AI-powered simulations enhance DM preparedness and training.

Further integrating AI into EMS department training presents ethical and logistical concerns. Future research should focus on optimizing AI applications, ensuring ethical compliance and awareness, and improving accessibility for EMS departments with limited resources. AI should be embraced in EMS training and has the potential to greatly enhance provider competency, confidence, and ultimately, patient outcomes.

## Figures and Tables

**Figure 2:**
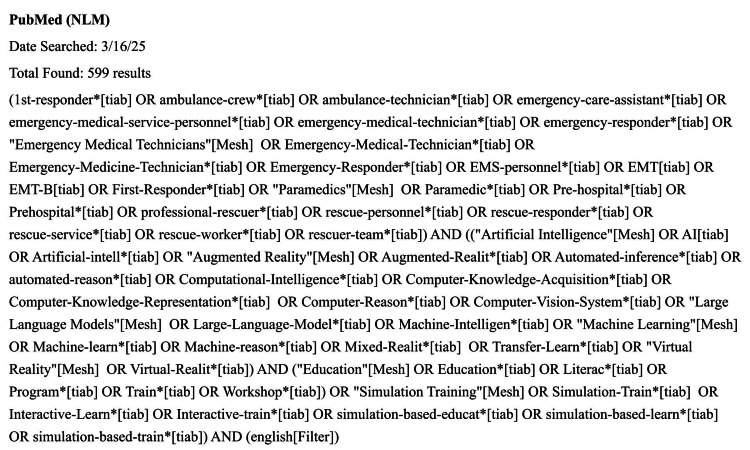
PubMed search terms

**Figure 3:**
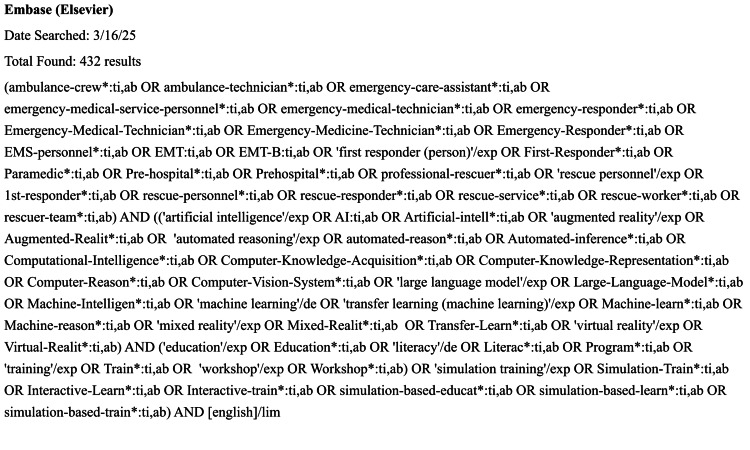
Embase search terms

**Figure 4:**
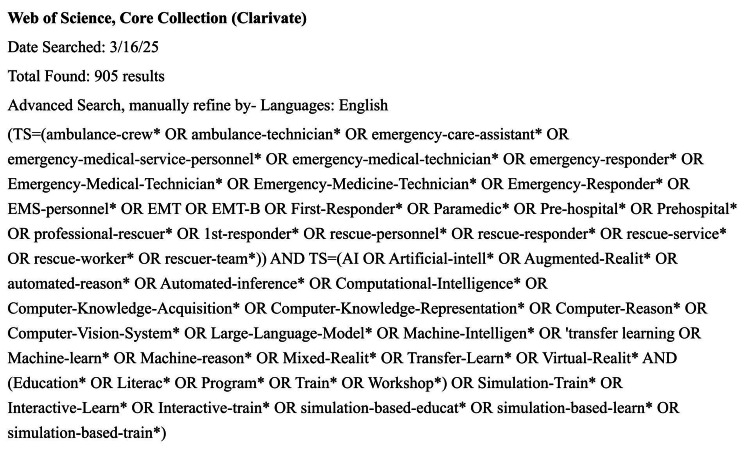
Web of Science search terms

## Appendices

Appendix A 

Appendix B

Appendix C
